# Patients admitted to treatment for substance use disorder in Norway: a population-based case–control study of socio-demographic correlates and comparative analyses across substance use disorders

**DOI:** 10.1186/s12889-022-13199-5

**Published:** 2022-04-20

**Authors:** Ellen J. Amundsen, Anne Line Bretteville-Jensen, Ingeborg Rossow

**Affiliations:** Department of Alcohol, Tobacco and Drugs, Norwegian Institute of Public Health, POB 222 Skøyen, 0213 Oslo, Norway

**Keywords:** Substance use disorder, Patients, Population control cohort, Socio-demographic correlates, Register linkage, Norway

## Abstract

**Background:**

Improved knowledge regarding socio-demographic correlates of people with substance use disorders (SUDs) is essential to better plan and provide adequate services for SUD patients and their families, and to improve our understanding of the complex mechanisms underlying progression into and development of various SUDs. This study aimed to: i) describe demographic, economic, and social correlates of people with SUDs in comparison with those of the general population and ii) compare these correlates across SUDs from licit versus illicit substances, as well as across specific SUDs.

**Methods:**

A national population-based case–control study included *all* SUD patients enrolled in specialized drug treatment in Norway in 2009–2010 (*N* = 31 245) and a population control sample, frequency-matched on age and gender (*N* = 31 275). Data on education level, labour market participation, income level and sources, and family/living arrangement were obtained by linkages to national registers.

**Results:**

Demographic, economic, and social correlates of SUD patients differed substantially from those of the general population, and across specific SUDs. Among SUD patients, those with illicit – as compared to licit – SUDs were younger (mean quotient = 0.72 [0.71–0.72]), more often had low education level (RR = 1.68 [1.63–1.73]), were less often in paid work (RR = 0.74 [0.72–0.76]) and had lower income (mean quotient = 0.61 [0.60–0.62]). Comparison of patients with different SUD diagnoses revealed substantial demographic differences, including the relatively low mean age among cannabis patients and the high share of females among sedatives/hypnotics patients. Opioid patients stood out by being older, and more often out of work, receiving social security benefits, and living alone. Cocaine and alcohol patients were more often better educated, included in the work force, and had a better financial situation.

**Conclusion:**

Findings revealed substantial and important differences in socio-demographic correlates between SUD patients and the general population, between SUD patients with illicit and with licit substance use, and across specific SUD patient groups.

**Supplementary Information:**

The online version contains supplementary material available at 10.1186/s12889-022-13199-5.

## Introduction

Substance use disorders (SUDs) are among the most common psychiatric disorders in high-income countries. They often result from long-term extensive use of one or several addictive substances, they are strongly co-morbid with other psychiatric and somatic diseases, they are not easily treatable, and they often place high economic and welfare burdens on their immediate social networks as well as society at large [1–4]. Thus, SUDs constitute a significant public health problem in several respects: i) by accounting for a substantial fraction of the global health burden [5], ii) by affecting the health and well-being of family and other close relations, and iii) by contributing to socioeconomic inequality in mortality [6, 7]. The latter reflects the fact that SUDs are typically more prevalent in low socio-economic status groups [8, 9]. In the present study, we examine the demographic, economic and social correlates of people with substance use disorders and how these correlates may vary by primary drug (main diagnosis at treatment entry). In the introduction, we motivate research on such socio-demographic correlates of people with substance use disorder (PWSUD), and we review previous literature and knowledge gaps.

In the epidemiological literature, socio-demographic correlates of PWSUD are often presented merely as background information in studies with another scope or focus. However, such correlates – and particularly how they vary across different substance user groups – are important in their own right. There are several reasons for this. First, more systematic insight into the demographic, economic and social correlates of PWSUD is important for planning and providing adequate services for them, as well as for their family members and other close relations. Second, it is also relevant for assessing the choice of measures and task force areas in strategies to reduce social inequality in alcohol- and drug-related health problems. Finally, it may contribute to better understanding of the complex mechanisms underlying progression into and continuance of various SUDs, as socio-demographic correlates may reflect contributing causal or mediating factors.

Current knowledge about socio-demographic correlates of PWSUD mainly stems from one of two types of study samples: general population samples and SUD patients in treatment [10, 11], which both have important limitations. Large population surveys provide important knowledge about socio-demographic correlates of PWSUD compared to the general population, but mainly for the more common types of SUD, including alcohol use disorder (AUD) and cannabis use disorder (CUD) (e.g. [8, 10, 12, 13]). Moreover, they are inherently limited in their capacity to differentiate between various drug use disorders (DUD)[13]. Another limitation with general population surveys is that survey participants who report extensive substance use, may not be representative of the group as such – PWSUD may be harder to reach and less likely to participate when approached [14]. Treatment samples, on the other hand, often include patients who have one specific SUD, such as heroin addiction, crack cocaine addiction or alcohol dependence (e.g. [15–18]). Thus, these studies preclude comparisons of socio-demographic correlates across specific SUDs. Moreover, they generally provide limited information about the socio-demographic characteristics of SUD patients. With these limitations noted, the literature suggests that the prevalence of SUDs overall is elevated among males and younger adults [10, 11, 13, 19], among non-married individuals and among those with low education or/and income level [8–10]. Even when considering all SUDs, findings are mixed, which may reflect insufficient comparability across study methods, and socio-demographic correlates of SUDs seem to vary across jurisdictions and substance use cultures [11]. Moreover, the literature is sparse with regard to certain socio-demographic correlates, including urban dwelling [10].

Few previous studies have, apparently, examined the socio-demographic correlates of PWSUD in treatment across different substance user groups. Through extensive literature searches, we identified only four such studies [20–23] in adult populations. One study [20] distinguished between three groups of SUDs: AUD, CUD and opioid use disorder, whereas the remaining three [21–23] distinguished only between AUD and DUD. In these studies, correlates of SUDs from stimulant drugs or sedatives/hypnotics were not examined specifically in relation to other SUDs. Thus, there seems to be little knowledge about socio-demographic correlates across a range of specific SUDs.

In the present study, we applied another approach to describing the socio-demographic correlates of PWSUD, by employing a population-based case–control design and including a broad range of socio-demographic correlates from national registers. With this approach, we overcome the above-noted limitations pertaining to large population surveys and most previous treatment sample studies. Moreover, Norway may be particularly well suited for such a study, as SUD treatment is part of the specialized public health services, which is offered basically free of charge to the patients. Thus, in this setting, any social or economic characteristics of PWSUD are less likely to reflect treatment barriers due to treatment costs.

We highlight the issue of extensive use of sedatives/hypnotics (e.g., benzodiazepines), which is often overlooked by policymakers and the scientific community [24–26]. While population surveys suggest that the prevalence of extensive use/misuse or dependence of benzodiazepines is relatively high compared to most other drugs [26], treatment rates are very low, especially in addiction service centres [27]. For this group of SUDs, demographic correlates are described in population surveys, where prevalence of ‘extensive’ use (which encompasses a broader category of users than those fulfilling criteria for DUD) is highest in young adults and equally distributed by gender [26]. In this comparative study of PWSUD in Norway, patients with sedatives/hypnotics dependence are also included, and we may thus provide a broader and more nuanced picture of the socio-demographic correlates of PWSUD with respect to various licit versus illicit psychotropic substances.

The aim of this study was to describe socio-demographic correlates of people admitted to treatment for SUD and to compare these correlates to those of the general population. Moreover, we compared the socio-demographic correlates of patients with licit SUDs to those with illicit SUDs, and finally, we compared these correlates across SUDs.

## Methods

### Design and participants

We employed a population-based case–control design [28] to compare socio-demographic characteristics between SUD patients and the general population. Data on SUD patients (cases) were obtained from the Norwegian Patient Registry, which covers the entire population of patients in the publicly financed specialized healthcare system in Norway. The patient sample encompasses all those who were admitted to specialized SUD treatment or to a psychiatric hospital in 2009 or 2010 with a SUD main diagnosis, that is ICD-10 diagnoses F10 (alcohol) (*N* = 12 448), F11 (opioids) (*N* = 5860), F12 (cannabis) (*N* = 3584), F14 (sedatives and hypnotics) (*N* = 1466), F15 (cocaine) (*N* = 197), F16 (other stimulants) (*N* = 2354) or F19 (other or several substances, which may include alcohol, sedatives or hypnotics) (*N* = 5336) (total *N* = 31 245). For the sake of brevity, in the following, we will refer to patients with main diagnosis F10 as “alcohol patients”, patients with main diagnosis F11 as “opioid patients” etc. In addition to the abbreviation PWSUD, we use PWAUD (people with alcohol use disorder) and PWDUD (people with drug use disorder).

A sample of controls was provided by Statistics Norway. This was randomly drawn from the Norwegian National Population Register on 1 January 2010 (midpoint of the patient recruitment period) and frequency-matched with the SUD patient sample by birth year and gender (*N* = 31 275). In other words, the control group was sampled from the entire source population that gave rise to the cases, and with this ‘inclusive design’ the control group will include some cases [29]. In our study, 353 patients happened by chance to also be in the control sample, and these were included in the study as both cases and controls.

Using unique national ID numbers, the data from the patient sample and the control cohort were linked to national administrative registries for the whole population in order to obtain individual data on socio-demographic correlates.

### Measures

For most socio-demographic correlates, we employed information registered the year before study entry.

Year of birth and gender were obtained from Statistics Norway. Age was calculated as year of study entry minus year of birth. Gender (legal) was operationalized using the third digit of the national ID number at the time of inclusion in the study. Legal gender can be changed after birth.

Own and the father’s completed education were extracted from the National Education Database (NUDB). Cut-off for dichotomous variables separated those with low education level (completed mandatory education, i.e., 7, 9 or 10 years depending on birth cohort) from others. The father’s completed education level was fixed at age 16 of the study participants (data extracted from NUDB).

On the basis of data obtained from the Income Registry, Statistics Norway, variables on labour market participation (“in paid work”), total income and wealth were constructed. Income from paid work included income from wages plus net business income, dichotomized into larger than zero vs. not larger than zero. Total income was defined as the sum of income from paid work, capital income, taxable and tax-free transfers during the calendar year. Fixed tax and other negative transfers were not deducted. Wealth was defined as the sum of calculated real capital and calculated gross financial capital. Gross financial capital includes bank deposits, units in equity, bond and money market funds, shares, assets in share savings accounts, bonds, and other securities. A three-year average was employed for both income and wealth.

Data on two types of social security benefits were obtained from Statistics Norway and dichotomized into receiving the benefit vs. not receiving the benefit. First, financial assistance is a temporary economic help to cover necessary expenses (e.g., for food, clothes, house rent, etc.). Second, disability pension is a permanent source of income, granted to persons with a minimum 50% permanent reduction of work capacity due to illness or injury. Disability pension can be granted to people with a drug dependence diagnosis.

Living alone (dichotomized into single-person household vs. other household size) and living with young children (dichotomized into families with child(ren) under six years of age vs. other family types) were extracted from the National Population Register for 1 January of the year of study entry.

Urban dwelling was obtained from the National Population Register and dichotomized as living in cities with more than 50 000 inhabitants vs. other city/ municipality sizes, as of 1 January 2010. Hence, data on urban dwelling is missing for patients who died in 2009 (*N* = 267).

### Missing data

The administrative registers were not entirely complete. The original sample included 31 281 patients and 31 281 controls. Age (year of birth) or sex were missing for 36 cases (0.1%) and 6 controls (0.02%), however. Only those with known age and sex were included in the analyses (see Table 1, 31 245 patients and 31 275 controls). Missing information for other measures totalled less than 3%, except for father’s education, where 13% of the sample had no registration. No imputation algorithm was employed.

**Table 1:** Patient^1^ and control^2^ cohort, by socio-demographic correlates

	**Patient cohort** **(*N* = 31 245)**	**Population cohort** **(*N* = 31 275)**	**Relative risk/ mean quotient**	***p*-value**^6^
	**Range (), 99% CI []**	**Range (), 99% CI []**	**99% CI []**	
**Demographic and social correlates**				
Age. Mean (range)	39.3 (14–93)	40.0 (14–94)	0.98 [0.98–0.99]	-^5^
Males. %	68.8 [68.1–69.4]	68.8 [68.1–69.4]	1.00 [0.99–1.01]	-^5^
Low education level. %	57.7 [56.9–58.4]	24.5 [23.9–25.2]	2.35 [2.28–2.42]	< 0.001
Father’s education (low level). %	42.1 [41.3–42.8]	33.2 [32.4–34.0]	1.27 [1.23–1.30]	< 0.001
Living alone. %	47.5 [46.8–48.3]	20.9 [20.3–21.5]	2.27 [2.20–2.35]	< 0.001
Living with young children. %	7.0 [6.7–7.4]	17.7 [17.2–18.3]	0.40 [0.37–0.42]	< 0.001
Urban dwelling. %	44.7 [44.0–45.5]	38.4 [37.7–39.1]	1.16 [1.14–1.19]	< 0.001
**Economic correlates**				
In paid work^3^. %	52.7 [51.9–53.4]	89.7 [89.2–90.1]	0.59 [0.58–0.60]	< 0.001
Total income. Three years’ mean. 1000 NOK	224 [222–226]	378 [373 -382]	0.59 [0.58–0.60]	< 0.001
Wealth. Three years’ mean. 1000 NOK	182 [170 -193]	606 [569–643]	0.30 [0.27–0.33]	< 0.001
Disability pension^4^. %	18.1 [17.6–18.7]	5.8 [5.5–6.1]	3.12 [2.92–3.34]	< 0.001
Financial assistance/supplementary benefit. %	44.5 [43.8–45.2]	3.9 [3.6–4.2]	11.38 [10.60–12.29]	< 0.001

^1^ Substance use disorder patients with treatment admission 2009–2010. ^2^ Control cohort per 1 January 2010 with same age and gender distribution as the patient cohort. ^3^ Age group 18–66 years of age. ^4^ Granted to persons 18–66 years of age. ^5^ Matching variables. ^6^ With ten *p*-values less than 0.001, the probability of at least one significant result by chance will be less than 0.01

### Analyses

Socio-demographic correlates were compared between three sets of groups; i) between patients and controls; ii) between patients with illicit versus licit substance use, and iii) between patients with specific SUDs. Confidence intervals of relative risks in Tables 1 and 2 were calculated using MedCalc’s statistical calculator [30], while confidence intervals of mean quotients were calculated using GraphPad’s Quick Calcs [31]. STATA 16 was used for other calculations.

**Table 2:** Patients^1^ with illicit and licit substance use disorders, by socio-demographic correlates

	**Illicit substance use disorder**^**2**^ **(*N* = 17 331)**	**Licit substance use disorder**^**2**^ **(*N* = 13 914)**	**Relative risk/ Mean quotient**	***p*-value**^6^
	**Range (), 99% CI []**	**Range (), 99% CI [**	**99% CI []**	
**Demographic and social correlates**				
Age. Mean (range)	33.4 (15–93)	46.6 (14–88)	0.72 [0.71–0.72]	< 0.001
Males. %	69.6 [68.7–70.5]	67.6 [66.6–68.7]	1.03 [1.01–1.05]	< 0.001
Low education level. %				
Unadjusted	70.4 [69.5–71.3]	41.9 [40.8–43.0]	1.68 [1.63–1.73]	< 0.001
Adjusted^3^	65.4 [64.2–66.6]	49.2 [48.0–50.4]	1.33 [1.29–1.36]	< 0.001
Father’s education (low level). %				
Unadjusted	40.6 [39.6–41.6]	44.0 [42.8–45.2]	0.92 [0.89–0.96]	< 0.001
Adjusted^3^	43.0 [41.8–44.2]	40.8 [39.5–42.2]	1.05 [1.01–1.10]	< 0.001
Living alone. %				
Unadjusted	48.8 [47.8–49.5]	46.0 [44.9–47.1]	1.06 [1.03–1.09]	< 0.001
Adjusted^3^	52.8 [51.6–54.0]	43.7 [42.5–44.9]	1.21 [1.17–1.25]	< 0.001
Living with young children. %				
Unadjusted	7.6 [7.1–8.1]	6.3 [5.7–6.8]	1.21 [1.09–1.36]	< 0.001
Adjusted^3^	6.2 [5.8–6.7]	8.4 [7.6–9.1]	0.74 [0.66–0.83]	< 0.001
Urban dwelling. %				
Unadjusted	46.1 [45.1–47.1]	43.0 [41.9–44.1]	1.07 [1.04–1.11]	< 0.001
Adjusted^3^	47.3 [46.1–48.5]	42.9 [41.2–44.1]	1.10 [1.06–1.15]	< 0.001
**Economic correlates**				
In paid work^4^. %				
Unadjusted	45.6 [44.7–46.6]	61.7 [60.6–62.8]	0.74 [0.72–0.76]	< 0.001
Adjusted^3^	41.2 [40.0–42.3]	64.7 [63.5–66.0]	0.64 [0.62–0.66]	< 0.001
Total income. Three years’ mean. 1000 NOK				
Unadjusted	175 [174 -176]	285 [282 -289]	0.61 [0.60–0.62]	< 0.001
Adjusted^3^	192 [188 -196]	256 [254 -259]	0.75 [0.73–0.77]	< 0.001
Wealth. Three years’ mean. 1000 NOK				
Unadjusted	71 [59 -83]	320 [299 -340]	0.22 [0.18–0.26]	< 0.001
Adjusted^3^	114 [93 -136]	232 [216 -7]	0.49 [0.40–0.59]	< 0.001
Disability pension^5^. %				
Unadjusted	14.6 [13.9–15.3]	22.7 [21.8–23.7]	0.64 [0.60–0.68]	< 0.001
Adjusted^3^	24.8 [23.8–25.8]	15.2 [14.6–15.9]	1.63 [1.53–1.73]	< 0.001
Financial assistance/supplementary benefit. %				
Unadjusted	58.8 [57.9–59.8]	26.6 [25.7–27.6]	2.21 [2.12–2.30]	< 0.001
Adjusted^3^	57.3 [56.1–58.4]	30.7 [29.5–31.9]	1.87 [1.79–1.95]	< 0.001

^1^ Substance use disorder patients with treatment admission 2009–2010. ^2^ Licit SUD includes alcohol use disorder and sedatives/hypnotics disorder, while illicit includes opioid, cannabis, and stimulant disorders, as well as disorders including several or other drugs. ^3^ Adjusted to the gender and age distribution among all patients in the study. ^4^ Age group 18–66 years of age. ^5^ Granted to persons 18–66 years of age. ^6^ With eleven p-values less than 0.001, the probability of at least one significant result by chance will be less than 0.01

As controls were frequency-matched for year of birth and gender, the differences between the patients and controls were automatically adjusted for age and gender. Differences between groups of patients are provided as raw figures to show actual group correlates. In addition, figures are shown adjusted for age and gender (direct standardized values) to the age and gender distribution in the whole patient population to illustrate the impact of these effect modifiers.

Bonferroni corrections were carried out for hypotheses regarding differences between SUD patients and the population, as well as between SUD patients admitted to treatment dependent of illicit vs. licit drugs. We set the level of statistical significance to 1% and calculated 99% confidence intervals which gives a certain protection towards false positive findings without reducing the statistical power. Comparisons of the seven groups of SUD patients were carried out more pragmatically based only on non-overlapping 99% CI.

## Results

The SUD patients differed substantially from the population control cohort on all socio-demographic correlates except for the matching variables age and gender (Table 1). Thus, compared to the control cohort, a larger proportion of the SUD patients had low education level (57.7% vs. 24.5%), and a larger proportion of their fathers had a low education level (42.1% vs. 33.2%). The SUD patients were more often living in single-person households (47.5% vs. 20.9%), were less likely to live in a family with young children (7.0% vs. 17.7%), and were more likely to live in an urban area (44.7% vs. 38.4%). Moreover, SUD patients were less likely to be in paid work (52.7% vs. 98.7%), they had lower personal income (NOK 224 000 vs. 378 000) and wealth (NOK 182 000 vs. 606 000), and they were more likely to receive social security benefits (disability pension 18.1% vs. 5.8%, financial assistance 44.5% vs 3.9%).

In the following, we focus on patients only. Table 2 presents the corresponding correlates and compares patients with SUDs from illicit substances (opioids, cannabis, cocaine, other stimulants, or “several or other drugs”) to patients with SUDs from licit substances (alcohol or hypnotics/sedatives). Overall, those with SUDs from illicit drug use were considerably younger (mean age 33.4 years vs. 46.6 years), they were more likely to have a low education level (70.4% vs. 41.9%), were less likely to be in paid work (45.6% vs. 61.7%), had lower income (NOK 175 000 vs. 285 000) and wealth (NOK 71 000 vs. 320 000), were less likely to receive disability pension (14.1% vs. 22.7%), and more likely to receive financial assistance (58.8% vs 26.6%), compared to their licit drug use counterparts.

**Fig. 1:**
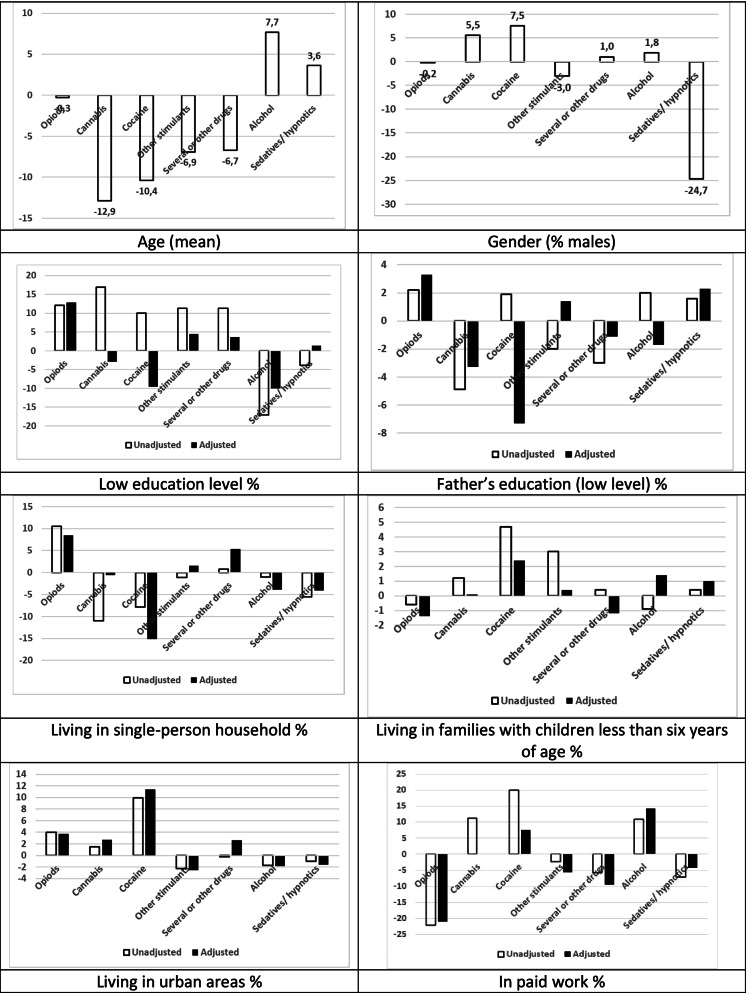
Patients^1^ by main diagnosis^2^ and socio-demographic correlates. Percentage point difference from mean value for all patients. Unadjusted and adjusted^3^. Legend: ^1^Substance use disorder patients with treatment admission 2009–2010. ^2^Main diagnosis by ICD 10. ^3^Adjusted to the gender and age distribution among all patients in the study

**Fig. 2:**
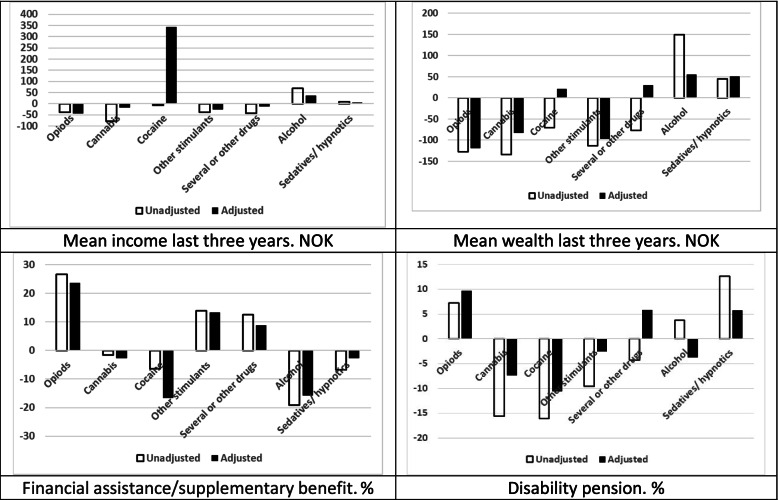
Patients^1^ by main diagnosis^2^ and economic correlates. Percentage point and NOK difference from mean value for all patients. Unadjusted and adjusted^3^. Legend: ^1^Substance use disorder patients with treatment admission 2009–2010. ^2^Main diagnosis by ICD 10. ^3^Adjusted to the gender and age distribution among all patients in the study

Next, we compared socio-demographic unadjusted correlates across specific SUD categories. We illustrated the differences by presenting for each specific SUD the relative deviation (in per cent) from the mean for all patients. Among patients with SUDs from illicit substances, opioid patients stood out by being older, less often in paid work, and more often receiving social security benefits, and living alone (Figs. 1 and 2. See also supplementary Tables S1 and S2 which include means for each SUD with confidence intervals). The cocaine patients, on the other hand, more often had paid work and higher income. Cannabis patients were the youngest and had a low level of education. In addition, both cocaine and cannabis patients included more males than the other SUD patients, had a high level of workforce participation and a low level of receiving social security benefits. Among patients with SUDs from licit substances, alcohol patients, in comparison with sedatives/hypnotics patients, were less often of low education level (40.5% vs. 53.8%), were more often in paid work (63.6% vs. 45.6%), had higher income (NOK 291 000 vs. 231 000) and wealth (NOK 331 000 vs. 227 000) and were less often in receipt of financial assistance (25.4% vs. 37.2%) and disability pension (21.8% vs. 30.7%). They more often lived alone (46.5% vs. 41.9%), and a higher proportion were male (70.4% vs. 42.9%) (see Figs. 1 and 2 and Supplementary Tables S1 and S2, for more details).

Adjustment for age and gender amplified, attenuated, or even reversed the unadjusted differences in socio-demographic correlates across patient groups (Figs. 1 and 2). We observed the largest differences between unadjusted and adjusted deviations with regard to own education level and father’s education level.

## Discussion

This study found that, compared to the general population, the SUD patients had on average substantially lower education level, income, and wealth, they were less often in paid work and more often recipients of social security benefits. Among SUD patients, users of illicit substances scored worse on all socio-economic status indicators, compared to users of licit substances. Comparison of patients with different SUD diagnoses further revealed important differences within and across patient groups. Demographic differences, such as the relatively low mean age among cannabis patients or the high share of females among sedatives/hypnotics patients should be noted, as should the large differences in education level, labour market participation, income and wealth across specific SUD patient groups. These are all factors of probable importance for treatment outcome and future welfare and economic independence.

This study is likely the first to report on demographic, economic and social correlates of a complete national SUD patient cohort and to compare a broad range of correlates across patients with different SUDs, including sedatives/hypnotics patients. While there are few previous studies that resemble our study in reporting socio-demographic correlates for various specific SUDs, our findings corroborate previous findings in respect of correlates of PWSUD [8, 9, 18, 32, 33]. Thus, compared to the general population, SUD patients in our study were more likely to live alone, have low education, and low income. Moreover, our findings accord with previous studies with regard to comparisons between people with AUD and DUD, finding that the former group is characterized by higher age [20, 22, 23], higher proportion in paid work [22, 23] and higher income [22]. Focusing on sedatives/hypnotics patients specifically, we found that, compared to other SUD patients, these were older and more often women, whereas, for most other socio-demographic correlates, they did not deviate from other patients. A recent review of the epidemiology of sedatives/hypnotics misuse (i.e. benzodiazepines) showed that the literature is mixed, with no clear pattern of socio-demographic correlates of this group [26]. These mixed findings may, however, reflect the fact that the primary studies included in the review were based on heterogenous samples, including patients in SUD treatment and PWSUD in general population samples.

We found substantial differences between unadjusted and adjusted differences in socio-demographic correlates across patient groups. This observation reflects the fact that age and gender distributions varied across SUD groups and were associated with the other examined socio-demographic variables. Adjusted figures present what the variation across SUD groups would look like if all groups had had the same age and gender distribution.

What can explain the observed socio-demographic differences between PWSUD and the general population and between substance-specific groups of PWSUD? Overall, it seems likely that the underlying mechanisms are complex, and, in the following, we will briefly discuss three possible main pathways.

The first pathway pertains to socio-demographic selection mechanisms into extensive or problematic substance use. With regard to alcohol and illicit drugs, socio-demographic differences are more prominent when use has evolved to extensive use, and this occurs more often among males and in low socio-economic status groups [19]. On the other hand, extensive use of sedatives/hypnotics is equally or more often seen in women [26]. Coping with negative affect and sleeping problems are the most common motives for benzodiazepine misuse [26], and also more prevalent among women and older adults [34] which, at least in part, may suggest that extensive use more often develops in these population groups. Moreover, falling outside education or employment seems to increase the risk of developing a SUD [35, 36]. It is well established that impulsivity, or poor inhibitory control, is a precursor for SUD vulnerability [37], and as impulsivity is also associated with low academic achievement [38], this individual correlate may, in part, explain the association between low education level and SUD. Moreover, impulsivity occurs more frequently in PWDUD compared to PWAUD [39], which may partly explain the higher share of low education level in the former group. This finding may however also be partly explained by a more rapid development of dependency for some illicit drugs than for alcohol [40], which could imply that a larger proportion of PWDUD develops a substance use problem while the user is still in education. Further, the higher education level, labour market participation and the overall better financial situation for PWAUD in particular may in turn delay treatment seeking for this group. The tendency for those with SUDs from illicit drugs to live in urban areas may, in part, reflect easier access to illegal drugs at a lower price, and a possible influx of drug users from rural areas.

The second pathway pertains to socio-demographic differences in the utilization of SUD treatment services. Studies from the US and European countries have reported higher likelihood of specialized treatment utilization among those with low income and those with psychiatric co-morbidity [41, 42]. Other factors that seem to increase treatment-seeking include younger age and lower education level [42]. However, compared to PWAUD, PWDUD are more likely to seek treatment shortly after disorder onset [42]. With regard to PWAUD, few seek treatment on their own: around half remain undiagnosed if doctors only rely on their clinical judgement, and diagnosis and referral to SUD treatment most likely occur when the patient is treated for other medical problems, including alcohol-related injury or liver disease [43]. Thus, the first treatment episode is typically delayed until the disorder is well established and more severe [43], which is well in line with our observation of higher age among AUD patients compared to other SUD patients. Moreover, while women are generally more likely than men to seek treatment in general health and mental health care services, some studies found that women are less likely to seek treatment for alcohol problems [44, 45]. We found an elevated likelihood of urban dwelling among SUD patients. This may possibly be explained by easier access to treatment services, even in a treatment system with universal coverage, as in Norway. As impulsivity is associated with lower academic achievement and also occurs more frequently among PWDUD than among PWAUD, differential treatment utilization by PWAUD and PWDUD may contribute to the higher proportion of low education level among patients with illicit SUDs compared to other SUD patients.

The third pathway pertains to the social drift reflecting consequences of extensive substance use. People with SUDs are at elevated risk of early retirement, unemployment, low income and need of social assistance [46, 47]. Further, SUDs affect the ability to commit in family life and they are associated with family dysfunction and child abuse and neglect [48], which increases the likelihood of marital breakdown or/and loss of parental custody. This corrobrates our finding that SUD patients more often lived alone and less often lived in a family with young children. One element in this pathway is the preoccupation with accessing and using the substance [49] at the cost of other activities, including education and family life commitment. Another element is the cognitive impairment due to SUD [50], which in turn impacts capacity for education and employment. The impact of SUDs on unemployment and financial difficulties seems to be larger for PWDUD compared to PWAUD [47], which corroborates our findings. Substance-related discrimination in the labour market may also account for some of the higher unemployment and financial difficulties among SUD patients. Thus, among full-time employed people, SUD seems to be a risk factor for involuntary job loss, and more so among PWDUD than PWAUD [51]. Moreover, the high financial costs of extensive substance use, and particularly so for illicit drugs due to elevated retail prices in illegal markets [52], may be difficult to cover with legal means. Studies of income-generation of PWSUD have revealed that many rely mainly on illegal income sources [53, 54], and this may partially explain the lower participation rate in legal employment among PWSUD from illicit, as compared to licit, substances.

Although we cannot determine which of these three pathways, or what combination of them, are at work here, improved knowledge of the socio-demographic correlates of SUD patients is warranted for several purposes. More exact knowledge regarding socio-economic patient correlates may help to better design, implement and evaluate drug treatment services to improve outcome and the potential for future individual welfare and economic independence. For instance, opioid patients with a high level of marginalization and a low level of own resources are in need of far more societal efforts to reach a stable situation than cocaine and alcohol patients who are better educated, included in the work force and with a better financial situation. Cannabis patients also have a low level of education and economic means, although they are younger than opioid patients and thus have fewer drug consuming years. These and other observed differences between patients in respect of gender, age, education, income (including stable income like disability pension and short-term income like supplementary benefits) and labour market participation may be taken into account when designing treatment programs for subgroups of PWSUD. They may also help explain differences in treatment outcomes across patient groups. Better knowledge of patient characteristics may further improve measures aimed at reducing social inequality related to SUD, as it may reveal for which subgroups inequality is particularly distinctive. Further, the complex mechanisms underlying progression into, and development of, various specific SUDs are currently not well understood. Using linked administrative datasets of national cohorts of SUD patients to describe and compare a range of demographic, economic and social correlates may contribute to improved understanding of selection mechanisms and causal associations, as suggested by the possible pathways discussed above.

### Limitations

While register-based data, as employed here, are not hampered by the typical limitations found in survey studies (selection and attrition bias, response biases and few observations of PWSUD), register data come with other limitations. Routine data may vary in data quality, coding may differ between persons and institutions, and it is often difficult to gain exact information on how such data were generated [55]. In our context, we do not know precisely what underlies the making of a specific SUD diagnosis, and as co-use of several substances is typical in PWSUD [56], it is possible that a single substance diagnosis (e.g. alcohol use disorder) may be somewhat arbitrarily assigned. This type of misclassification, however, is relevant only if it is systematic and correlated with socio-demographic correlates, which cannot be precluded. In our data set, we could not differentiate between opioid patients who were users of illegally obtained opioids (e.g. heroin) and opioid patients who were users of legally prescribed opioids. However, we assumed that the vast majority of opioid patients belonged to the former group, as problematic opioid use from prescription opioids seemed quite rare in Norway at the time of the study [57]. Further, our findings reflect a cross-sectional ‘snap-shot’ of patients in treatment for SUDs. Thus, time dynamics, for instance with regard to changes in substance use careers and changes in social drift, could not be assessed in this study. Finally, transferability of our findings to other settings may depend on the extent to which they differ from the Norwegian setting, for instance when there are substantial differences in prevalence of substance use, the type of treatment system and treatment coverage, and the type of welfare schemes.

Adjustment for age and gender amplified, attenuated, or even reversed the unadjusted differences in socio-demographic correlates across patient groups. This complexity is noteworthy in terms of understanding likely underlying mechanisms and for external validation of findings from other patient populations.

## Conclusion

This study revealed that demographic, economic and social correlates differ substantially between SUD patients and the general population. They also differ substantially between SUD patients with illicit versus licit substance use, and they differ across specific SUD patient groups. A broadened and more nuanced insight into socio-demographic correlates of patients with substance use disorder is important to inform the planning and provision of adequate treatment and care services for these vulnerable patients and their families, as well as to better understand the complex mechanisms underlying progression into and development of various substance use disorders.

## Supplementary Information

**Additional file 1: Table S1.** Patients^1^ by main diagnosis and socio-demographic correlates. **Table S2.** Patients^1^ by main diagnosis and economic correlates.

## Data Availability

The data that support the findings of this study are available from the Norwegian Patient Registry and registries in Statistics Norway. The data were linked and kept at the Norwegian Institute of Public Health. The linked data were used under licence for the current study, and are not publicly available. Data are however available for approved scientists upon reasonable request and with permission of Regional Committees for Medical and Health Research Ethics, as well as the registries included. In this regard, contact the co-author of this paper, Anne Line Bretteville-Jensen.

## Abbreviations

AUD: Alcohol use disorder
CUD: Cannabis use disorder
DUD: Drug use disorder
NUDB: National Education Database
PWAUD: People with alcohol use disorder
PWDUD: People with drug use disorder
PWSUD: People with substance use disorder
SUD: Substance use disorder
