# Supplementary material for: Patients admitted to treatment for substance use disorder in Norway: a population-based case–control study of socio-demographic correlates and comparative analyses across substance use disorders
Source: BMC Public Health. 2022 Apr 20;22:792. doi: 10.1186/s12889-022-13199-5 (PMC9020072; doi:10.1186/s12889-022-13199-5)
Supplement: Supplementary file 1 — Additional file 1: Table S1. Patients1 by main diagnosis and socio-demographic correlates. Table S2. Patients1 by main diagnosis and economic correlates. [file 12889_2022_13199_MOESM1_ESM.docx]

**Supplementary tables** **to manuscript:** **Patients admitted to treatment for substance use disorder in Norway: a population-based case-control study of socio-demographic correlates and comparative analyses across substance use disorders**

**Table S1.** **Patients^1^ by main diagnosis and socio-demographic correlates**

|  | Main diagnosis^2^ | | | | | | | |
| --- | --- | --- | --- | --- | --- | --- | --- | --- |
|  | Illicit substances | | | | |  | Licit substances | |
|  | Opioids  F11  (N=5860]  Range (), 99% CI [] | Cannabis  F12  (N=3584)  Range (), 99% CI [] | Cocaine  F14  (N=197)  Range (), 99% CI [] | Other stimulants  F15  (N=2354)  Range (), 99% CI [] | Several or  other drugs  F19  (N=5336)  Range (), 99% CI [] |  | Alcohol  F10  (N=12 448)  Range (), 99% CI [] | Sedatives/  hypnotics F13  (N=1466)  Range (), 99% CI [] |
|  |  |  |  |  |  |  |  |  |
| Age. Mean (range) | 39.0  (17-93) | 26.4  (15-67) | 28.9  (19-64) | 32.4  (17-68) | 32.6  (15-87) |  | 47.0  (14-88) | 42.9  (15-87) |
| Age (CI of mean) | [38.7-39.3] | [26.1-26.8] | [27.6-30.2] | [32.0-32.9] | [32.2-32.9] |  | [46.7-47.3] | [42.1-43.8] |
|  |  |  |  |  |  |  |  |  |
| Males. % | 68.4  [67.2-69.9] | 74.1  [72.3-76.0] | 76.1  [68.3-84.0] | 65.6  [63.1-68.1] | 69.6  [68.0-71.2] |  | 70.4  [69.4-71.5] | 43.9  [40.6-47.3] |
| Low education level. % |  |  |  |  |  |  |  |  |
| Unadjusted | 69.8  [68.2-71.4] | 74.6  [72.7-76.6] | 67.7  [58.9-76.6] | 69.0  [66.6-71.5] | 69.0  [67.4-70.7] |  | 40.5  [39.4-41.7] | 53.8  [50.4-57.2] |
| Adjusted^3^ | 70.5  [68.8-72.2] | 54.8  [50.1-59.5] | 48.2  [41.9-54.5] | 62.1  [58.7-65.6] | 61.3  [59.2-63.4] |  | 47.7  [46.4-49.0] | 59.1  [55.7-62.5] |
| Father’s education (low level). % |  |  |  |  |  |  |  |  |
| Unadjusted | 44.3  [42.5-46.1] | 37.2  [35.1-39.4] | 44.0  [34.2-53.9] | 40.1  [37.4-42.8] | 39.1  [37.3-40.9] |  | 44.1  [42.8-45.3] | 43.7  [40.1-47.3] |
| Adjusted^3^ | 45.4  [43.3-47.5] | 39.8  [34.7-44.9] | 34.8  [24.6-45.0] | 43.5  [40.1-46.9] | 41.0  [38.8-43.3] |  | 40.4  [39.0-41.9] | 44.4  [40.3-48.5] |
| Living alone. % |  |  |  |  |  |  |  |  |
| Unadjusted | 58.0  [56.3-59.7] | 36.5  [34.4-38.6] | 39.6  [30.6-48.6] | 46.4  [43.7-49.0] | 48.3  [46.6-50.1] |  | 46.5  [45.3-47.6] | 41.9  [38.5-45.2] |
| Adjusted^3^ | 56.0  [54.1-57.9] | 46.9  [41.2-51.8] | 32.4  [25.8-39.0] | 49.0  [45.7-52.3] | 52.8  [50.6-54.9] |  | 43.6  [42.3-44.9] | 43.4  [39.6-47.2] |
| Living with young children. % |  |  |  |  |  |  |  |  |
| Unadjusted | 6.4  [5.6-7.3] | 8.2  [7.0-9.4] | 11.7  [5.8-17.6] | 10.0  [8.4-11.6] | 7.4  [6.4-8.3] |  | 6.1  [5.6-6.7] | 7.4  [5.7-9.2] |
| Adjusted^3^ | 5.6  [4.8-6.4] | 7.1  [5.6-8.5] | 9.4  [4.3-14.4] | 7.4  [6.1-8.7] | 5.8  [5.0-6.5] |  | 8.4  [7.6-9.3] | 8.0  [5.9-10.1] |
| Urban dwelling. % |  |  |  |  |  |  |  |  |
| Unadjusted | 48.7  [47.0-50.4] | 46.2  [44.0-48.3] | 54.6  [45.4-63.9] | 42.4  [39.7-45.0] | 44.5  [42.7-46.3] |  | 42.9  [41.8-44.1] | 43.7  [40.3-47.1] |
| Adjusted^3^ | 48.4  [46.4-50.4] | 47.4  [42.4-52.4] | 56.1  [49.7-62.6] | 42.2  [38.8-45.7] | 47.3  [45.2-49.5] |  | 42.9  [41.5-44.3] | 43.1  [39.3-47.0] |

^1^ Substance use disorder patients with treatment admission 2009-2010 ^2^ Main diagnosis by ICD 10 ^3^ Adjusted to the gender and age distribution among all patients in the study

**Table S2. Patients^1^ by main diagnosis and economic correlates**

|  | Main diagnosis^2^ | | | | | | | |  |  |  |
| --- | --- | --- | --- | --- | --- | --- | --- | --- | --- | --- | --- |
|  | Illicit substances | | | | |  | Licit substances | |  |  |  |
|  | Opioids  F11  (N=5860)  99% CI [] | Cannabis  F12  (N=3584)  99% CI [] | Cocaine  F14  (N=197)  99% CI [] | Other stimulants F15  (N=2354)  99% CI [] | Several or other drugs  F19  (N=5336)  99% CI [] |  | Alcohol  F10  (N=12 448)  99% CI [] | Sedatives/  Hypnotics F13  (N=1466)  99% CI [] |  |  |  |
| In paid work^3^. % |  |  |  |  |  |  |  |  |  |  |  |
| Unadjusted | 30.7  [29.2-32.3] | 64.0  [61.8-66.0] | 72.7  [64.4-80.9] | 50.3  [47.6-53.0] | 47.0  [45.2-48.8] |  | 63.6  [62.5-64.8] | 45.6  [42.2-49.1] |  |  |  |
| Adjusted^4^ | 31.8  [29.9-33.6] | 52.9  [47.9-57.8] | 60.2  [53.3-67.1] | 47.1  [43.9-50.3] | 43.2  [41.1-45.3] |  | 66.9  [65.6-68.2] | 48.4  [44.5-52.4] |  |  |  |
| Total income. Three years’ mean. 1000 NOK |  |  |  |  |  |  |  |  |  |  |  |
| Unadjusted | 184  [182-186] | 145  [142-148] | 215  [193-237] | 184  [180-189] | 179  [175-183] |  | 291  [288-295) | 231  [225-236] |  |  |  |
| Adjusted^4^ | 178  [175-180] | 205  [198-213] | 565  [357-772] | 197  [192-202] | 211  [199-224] |  | 260  [257-263] | 227  [219-234] |  |  |  |
| Wealth. Three years’ mean. 1000 NOK |  |  |  |  |  |  |  |  |  |  |  |
| Unadjusted | 54  [50-58] | 48  [41-56] | 112  [86-138] | 69  [61-77] | 105  [66-143] |  | 331  [309-353] | 227  [172-282] |  |  |  |
| Adjusted^4^ | 63  [57-68] | 99  [76-122] | 203  [171-236] | 85  [72-98] | 212  [129-295] |  | 237  [220-255] | 233  [146-320] |  |  |  |
| Disability pension^5^. % |  |  |  |  |  |  |  |  |  |  |  |
| Unadjusted | 25.3  [23.9-26.8] | 2.5  [1.8-3.1] | 2.0  [0.1-4.6] | 8.5  [7.0-10.0] | 13.8  [12.5-15.0] |  | 21.8  [29.8-22.8] | 30.7  [27.6-33.9] |  |  |  |
| Adjusted^3^ | 27.8  [26.6-29.1] | 10.7  [6.3-15.2] | 7.5  [0.8-14.1] | 15.5  [12.6-18.4] | 24.0  [22.2-25.9] |  | 14.3  [13.7-15.1] | 23.9  [21.1-26.6] |  |  |  |
| Financial assistance/Supplementary benefit. % |  |  |  |  |  |  |  |  |  |  |  |
| Unadjusted | 71.1  [69.6-72.7] | 42.9  [40.7-45.0] | 38.1  [29.1-47.0] | 58.4  [55.8-61.1] | 57.0  [55.3-58.8] |  | 25.4  [24.4-26.4] | 37.2  [34.0-40.5] |  |  |  |
| Adjusted^4^ | 68.2  [66.4-70.1] | 41.8  [37.3-46.3] | 27.9  [18.7-37.2] | 57.8  [54.4-61.2] | 53.4  [51.3-55.5] |  | 28.8  [27.5-30.1] | 41.8  [38.2-45.5] |  |  |  |
|  |  |  |  |  |  |  |  |  |  |  |  |

^1^ Substance use disorder patients with treatment admission 2009-2010 ^2^ Main diagnosis by ICD 10 ^3^ Age group 18-66 years of age ^4^ Adjusted to the gender and age distribution among all patients in the study  ^5^Granted to persons 18-66 years of age
